# Reductions in US life expectancy from COVID-19 by Race and Ethnicity: Is 2021 a repetition of 2020?

**DOI:** 10.1101/2021.10.17.21265117

**Published:** 2021-10-21

**Authors:** Theresa Andrasfay, Noreen Goldman

## Abstract

COVID-19 had a huge mortality impact in the US in 2020 and accounted for the majority of the 1.5-year reduction in 2020 life expectancy at birth. There were also substantial racial/ethnic disparities in the mortality impact of COVID-19 in 2020, with the Black and Latino populations experiencing reductions in life expectancy at birth over twice the reduction experienced by the White population. Despite continued vulnerability of the Black and Latino populations, the hope was that widespread distribution of effective vaccines would mitigate the overall impact and reduce racial/ethnic disparities in 2021. In this study, we use cause-deleted life table methods to estimate the impact of COVID-19 mortality on 2021 US period life expectancy. Our partial-year estimates, based on provisional COVID-19 deaths for January-early October 2021 suggest that racial/ethnic disparities have persisted and that life expectancy at birth in 2021 has already declined by 1.2 years from pre-pandemic levels. Our projected full-year estimates, based on projections of COVID-19 deaths through the end of 2021 from the Institute for Health Metrics and Evaluation, suggest a 1.8-year reduction in US life expectancy at birth from pre-pandemic levels, a steeper decline than the estimates produced for 2020. The reductions in life expectancy at birth estimated for the Black and Latino populations are 1.6-2.4 times the impact for the White population.

## Introduction

The staggering death toll in the US from COVID-19 has been well-documented: deaths attributed to COVID-19 in 2020 account for almost three-quarters of the 1.5-year reduction in period life expectancy at birth, reversing over 16 years of progress in mortality improvement (Arias et al., 2021). In a previous paper, we predicted that widespread availability of an effective vaccine would lessen the impact of COVID-19 on 2021 life expectancy compared with 2020, although life expectancy was unlikely to return to pre-pandemic levels (Andrasfay & Goldman, 2021a). Several highly effective vaccines have indeed been developed in record time, but relatively low vaccine coverage in the US, combined with the highly transmissible Delta variant of SARS-CoV-2, have led to a mortality surge of 2,000 deaths per day by early fall and a total number of COVID-19 deaths as of October 15, 2021 exceeding 90% of the 2020 total (Centers for Disease Control and Prevention, 2021; Kupferschmidt & Wadman, 2021). These sobering numbers combined with the younger age distribution of deaths in 2021 (see Figure 1) – resulting partly from higher vaccination rates among older individuals – suggest that the impact of COVID-19 on life expectancy in 2021 may be at least as large as that in the preceding year.

The disproportionate impact of COVID-19 on the survival of vulnerable populations has also received extensive attention: the Latino and Black populations experienced declines in life expectancy over twice as large as that for Whites (Arias et al., 2021). Risk factors for COVID-19 infection and mortality, such as crowded living conditions, frontline jobs with high exposure to infection and low pay, dependence on public transport, low access to quality healthcare, and high rates of select chronic conditions, still characterize these groups, suggesting continued racial/ethnic disparities in COVID-19 mortality (Figueroa et al., 2021; Goldman et al., 2021; Macias Gil et al., 2020; Maness et al., 2021; Rodriguez-Diaz et al., 2020). A strategically-targeted vaccine distribution had the potential to reduce racial/ethnic disparities in COVID-19 mortality in 2021 (Wrigley-Field et al., 2021), but many individuals faced barriers to vaccination in the early months, including difficulty scheduling vaccine appointments online, lack of transportation to vaccination sites, and lack of time off work to get vaccinated and recover from side effects (Feldman, 2021; Stone, 2021). The resulting inequitable vaccine distribution and uptake may have further exacerbated racial/ethnic disparities in COVID-19 mortality. However, as vaccines became more widely available in the later months of 2021, racial/ethnic differentials in vaccination rates decreased (Funk & Gramlich, 2021; Ndugga et al., 2021). By August 2021, differences in vaccination rates by political affiliation, religion, and rural/urban status exceeded racial/ethnic differences (Funk & Gramlich, 2021). Although these changing vaccination patterns may ultimately reduce the disparities in COVID-19 mortality in 2021 relative to those in 2020, it is now almost certain that life expectancy estimates for 2021 will continue to reflect a huge toll experienced by all groups.

In the current study, we extend our previous work by estimating the impact of COVID-19 on period life expectancy at birth and at age 65 in 2021 for the total population and for the White, Latino, and Black populations in the US. We present estimates for the partial year (through October 9, 2021) as well as for all of 2021 using projected numbers of COVID-19 deaths from the Institute of Health Metrics and Evaluation for the final months of the year.

## Methods

To estimate the impact of COVID-19 on 2021 period life expectancy with partial-year data on all-cause mortality, we employ life table techniques developed to estimate the impact on life expectancy of eliminating one or more causes of death (i.e., cause-deleted life tables; Chiang, 1968). In the present study, we assume that mortality conditions in 2021 would be equivalent to those observed pre-pandemic (i.e., had COVID-19 not occurred), and then estimate how the inclusion of COVID-19 deaths alters these mortality conditions. Specifically, we take the pre-pandemic life tables to be cause-deleted life tables in which COVID-19 has been eliminated and recover all-cause life tables for 2021 that incorporate COVID-19 mortality. This strategy has been used in previous studies to estimate the impact of COVID-19 on 2020 life expectancy (Andrasfay & Goldman, 2021a, 2021b; Castro et al., 2021; Heuveline & Tzen, 2021). Although this procedure does not incorporate excess mortality from causes other than COVID-19, our life expectancy at birth estimate of 1.3 years for the total US population based on this method (Andrasfay & Goldman, 2021b) was within 0.2-years of the official 1.5-year National Vital Statistics System (NVSS) estimate (Arias et al., 2021).

Provisional COVID-19 deaths by age, race, and ethnicity are provided by the National Center for Health Statistics (National Center for Health Statistics, 2021). These data include all deaths for which COVID-19 is listed as an underlying, probable, or presumed cause of death, and include deaths through October 9, 2021. The projected total number of COVID-19 deaths in the US by December 31, 2021, are taken from the October 15, 2021 projections issued by the Institute for Health Metrics and Evaluation (Institute for Health Metrics and Evaluation, 2021). At the time of writing, IHME published three scenarios for total reported COVID-19 deaths; we take the medium "current projection scenario" for our analyses. Because IHME projects cumulative COVID-19 deaths since the beginning of the pandemic, we subtract the total number of COVID-19 deaths reported by December 31, 2020. Mid-year 2021 population estimates by age, race, and ethnicity are obtained from the US Census Bureau (US Census Bureau, 2021). Life tables for 2018 for the total US population and for the non-Latino White, non-Latino Black, and Latino populations are obtained from the NVSS (Arias & Xu, 2020).[1]

We first estimate the expected number of deaths in 2021 in the absence of COVID-19 (${}_nD_{x,2021^*}$) by multiplying the 2018 age-specific mortality rates (${}_nM_{x,2018}$) by the 2021 mid-year population for the same age range (${}_nK_{x,2021}$). We then estimate the expected number of deaths in 2021 in the presence of COVID-19 (${}_nD_{x,2021}$) by adding the number of COVID-19 deaths in each age group (${}_nCOV_{x,2021}$) to the expected number of deaths in each age group from other causes. We assume that individuals who do not die of COVID-19 in 2021 are exposed to the 2018 mortality risks:

$$ {}_nD^*_{x,2021} = {}_nM_{x,2018} * {}_nK_{x,2021} $$
$$ {}_nD_{x,2021} = {}_nCOV_{x,2021} + {}_nM_{x,2018} * ( {}_nK_{x,2021} - {}_nCOV_{x,2021}) $$

[1] At the time of writing, 2018 was the most recent pre-pandemic year for which life tables were available separately by race and ethnicity; we will update these estimates when NVSS releases 2019 life tables. Life tables for other populations, including the Native American and Asian populations, are not published by NVSS at this time.

We then calculate the age-specific ratio of expected number of deaths in the absence of COVID-19 to expected number of deaths in the presence of COVID-19 (${}_{n}R_{x,2021}$). Using this ratio and Chiang's method (Chiang, 1968), we adjust the 2018 life table values to reflect the presence of COVID-19 mortality and obtain our estimates of all-cause life tables for 2021. We repeat these calculations for each of the racial/ethnic groups in our study.

Because total COVID-19 deaths in 2021 are still unknown at the time of writing (October 2021), we make two sets of calculations for 2021. The first set provides *partial-year* estimates that are based only on COVID-19 deaths reported to the NCHS through October 9, 2021. These calculations implicitly assume that there will be no additional COVID-19 deaths in 2021; they should be interpreted as an estimate of how COVID-19 deaths in 2021 *thus far* have impacted life expectancy and are a lower bound on the final impact in 2021. The second set provides estimates based on the total number of COVID-19 deaths projected by IHME under their "current projection scenario", which is their medium projection. Because IHME does not project deaths separately by age and race/ethnicity, we assume that future COVID-19 deaths in 2021 will have the same age and racial/ethnic distribution as deaths that have already occurred in 2021.

Because our cause-deleted life table methodology differs from that used by NVSS to estimate 2020 life expectancy, the magnitude of our 2021 estimates will not be directly comparable to the published 2020 life expectancy estimates from NVSS. To facilitate comparisons between 2020 and 2021, we also estimate 2020 life expectancy with the same methods as our 2021 calculations, using the provisional counts of COVID-19 deaths (rather than deaths from all causes) provided by NCHS for all of 2020, and mid-year 2020 population estimates provided by the US Census Bureau.[2]

## Results

Life expectancy estimates for the total US population and by race/ethnicity are displayed in Table 1. All reductions are relative to the 2018 life expectancy values, which are displayed in panel A at the top of this table. Our estimates of 2020 life expectancy reductions due to COVID-19 are displayed in panel B; these are estimated using cause-deleted methods, the same procedure used for the 2021 estimates. To highlight the comparisons between 2020 and 2021 under each of these scenarios, Figure 2 displays the magnitudes of the reductions in life expectancy at birth and at age 65 for the full-year 2020 estimates, partial-year 2021 estimates, and projected full-year 2021 estimates.

Panel C of Table 1 displays the partial-year 2021 estimates, based on the number of COVID-19 deaths through October 9, 2021. These estimates indicate that COVID-19 deaths through the first nine months of 2021 already imply a 1.2-year reduction in life expectancy at birth and a 0.8-year reduction in life expectancy at age 65 for the total US population. The reductions in life expectancy at birth are largest for the Latino population (2.4 years), followed by the Black population (1.5 years), and smallest for the White population (1.0 years). This reduction of one

[2] These 2020 estimates differ slightly from our previously published estimates (Andrasfay & Goldman, 2021b), because they include additional deaths that were not yet reported to NCHS at the time of the previous analysis, and they use 2020, as opposed to 2019, mid-year population counts.

year in life expectancy at birth for the White population already exceeds the 0.9-year reduction estimated for the full year in 2020. The reductions in life expectancy at birth for the Black and Latino populations are 0.4-0.5 years below those for 2020.

Panel D of Table 1 presents the 2021 estimates for a full-year based on the total number of reported COVID-19 deaths projected by IHME on October 15, 2021. Under this projection of 442,255 reported COVID-19 deaths, there would be a 1.8-year reduction in life expectancy at birth for the total US population, which substantially exceeds the 1.3-year estimated reduction for 2020 due to COVID-19. The 1.1-year reduction in life expectancy at age 65 for the total US population is equal to that estimated for 2020. These estimates for life expectancy at birth in 2021 *exceed* those for 2020 for all three racial/ethnic groups, with the difference between 2021 and 2020 reductions largest for the White population (an additional 0.4-year reduction). The estimated reductions in life expectancy at birth for the Latino population (3.1 years) and the Black population (2.1 years) are 2.4 and 1.6 times, respectively, the 1.3-year projected reduction for the White population. These disparities reveal another year of especially large racial/ethnic inequities underlying a large overall impact of COVID-19 on life expectancy.

## Discussion

Our preliminary estimates of period life expectancy suggest a devasting impact of COVID-19 in 2021, one that will probably be as large, if not larger, than that in 2020. The overall impact on life expectancy in 2021 will likely be greater than our projected estimates because our estimates incorporate only deaths from COVID-19. The cause-deleted life table methodology used in our analysis assumes that the risk of mortality from COVID-19 is independent of other causes of death, an assumption that is unlikely to be true. The effect of omitting other excess deaths is apparent from the NVSS estimates for 2020 life expectancy, which incorporate deaths from all causes. These reductions slightly exceed our estimates for 2020 based only on COVID-19 deaths because there were increases in several causes other than COVID-19 (e.g., unintentional injuries, homicides, diabetes) that were not offset by decreases in other causes (e.g., cancer, Alzheimer's disease, influenza, chronic lower respiratory diseases) between 2019 and 2020 (Arias et al., 2021; Glei, 2021).

These estimates also suggest continued large racial/ethnic disparities in the impact of COVID-19 on life expectancy. Although the estimates indicate some narrowing of the differentials from the previous year, this is entirely due to larger life expectancy reductions among the White population rather than to smaller decreases in either the Black or Latino populations. As with our 2020 estimates (Andrasfay & Goldman, 2021b), we project that the Latino population will experience the largest reduction in life expectancy at birth (3.1 years) in 2021 due to COVID-19. We project that the Black population will experience a 2.1-year reduction in life expectancy at birth in 2021 due to COVID-19, similar to the 1.9-year reduction we estimated for 2020 (Andrasfay & Goldman, 2021b).[3]

[3] The difference between our estimates based only on COVID-19 mortality and the NVSS estimates based on all-cause mortality were largest for the Black population (1.9 vs. 2.9 years), primarily because a higher proportion of 2020 deaths for the Black population, compared with Whites and Latinos, resulted from increases in non-COVID-19 causes (Arias et al., 2021; Luck et al., 2021). If all-cause mortality patterns are similar to those in 2020, it is likely that the ultimate reduction in 2021 life expectancy will be even larger for the Black population.

This analysis provides a preliminary set of estimates of the impact of COVID-19 on life expectancy in 2021 and its continued disparate impact on the Black and Latino populations, but it is subject to several limitations. As previously mentioned, the cause-deleted life table methodology does not account for excess mortality from causes other than COVID-19. Our partial-year estimates for 2021 life expectancy rely on NCHS provisional COVID-19 deaths, which are subject to reporting and processing delays, and so they are likely to be an underestimate of the impact of COVID-19 on life expectancy thus far in 2021. Our projected estimates for the full 2021 year rely on projected deaths for the remaining months of 2021. There is still much uncertainty regarding COVID-19 mortality in the final months of 2021, which will depend on further vaccine uptake, waning vaccine efficacy, and whether the US experiences another winter surge in cases, among other factors. We will continue to update these estimates as more data become available and as projections are updated.

**Table 1: Life expectancy estimates and reductions from 2018 for the total US population and by race/ethnicity**

| | **Total Population** | | **non-Latino White** | | **non-Latino Black** | | **Latino** | |
|---|---|---|---|---|---|---|---|---|
| | Birth | Age 65 | Birth | Age 65 | Birth | Age 65 | Birth | Age 65 |
| **A) Pre-pandemic** | | | | | | | | |
| 2018 $e_x$ | 78.7 | 19.5 | 78.6 | 19.4 | 74.7 | 18.0 | 81.8 | 21.4 |
| | | | | | | | | |
| **B) Full-year 2020 estimates** | | | | | | | | |
| Number of COVID-19 deaths | 385,256 | | 232,738 | | 61,473 | | 69,435 | |
| Estimated 2020 $e_x$ | 77.4 | 18.4 | 77.7 | 18.5 | 72.8 | 16.5 | 78.9 | 19.3 |
| Reduction from 2018 $e_x$ due to COVID-19 | -1.3 | -1.1 | -0.9 | -0.9 | -1.9 | -1.5 | -2.9 | -2.1 |
| | | | | | | | | |
| **C) Partial-year 2021 estimates** | | | | | | | | |
| Number of COVID-19 deaths through October 9, 2021 | 327,673 | | 207,410 | | 45,848 | | 57,077 | |
| Estimated 2021 $e_x$ | 77.5 | 18.7 | 77.6 | 18.7 | 73.2 | 17.1 | 79.4 | 19.9 |
| Reduction from 2018 $e_x$ due to COVID-19 | -1.2 | -0.8 | -1.0 | -0.7 | -1.5 | -0.9 | -2.4 | -1.5 |
| | | | | | | | | |
| **D) Projected full-year 2021 estimates** | | | | | | | | |
| Projected number of COVID-19 deaths in 2021 | 442,255 | | 280,416 | | 61,986 | | 77,168 | |
| Estimated 2021 $e_x$ | 76.9 | 18.4 | 77.3 | 18.4 | 72.6 | 16.7 | 78.7 | 19.4 |
| Reduction from 2018 $e_x$ due to COVID-19 | -1.8 | -1.1 | -1.3 | -1.0 | -2.1 | -1.3 | -3.1 | -2.0 |

Notes: Apart from life expectancy values from 2018 that are provided by the National Vital Statistics System, all life expectancy estimates are authors' calculations. Partial-year 2021 estimates and full-year 2020 estimates are based on provisional COVID-19 death counts provided by the National Center for Health Statistics (October 13, 2021 update). Projected full-year 2021 estimates are based on projected COVID-19 death counts through December 31, 2021, provided by the Institute for Health Metrics and Evaluation (October 15, 2021 update).

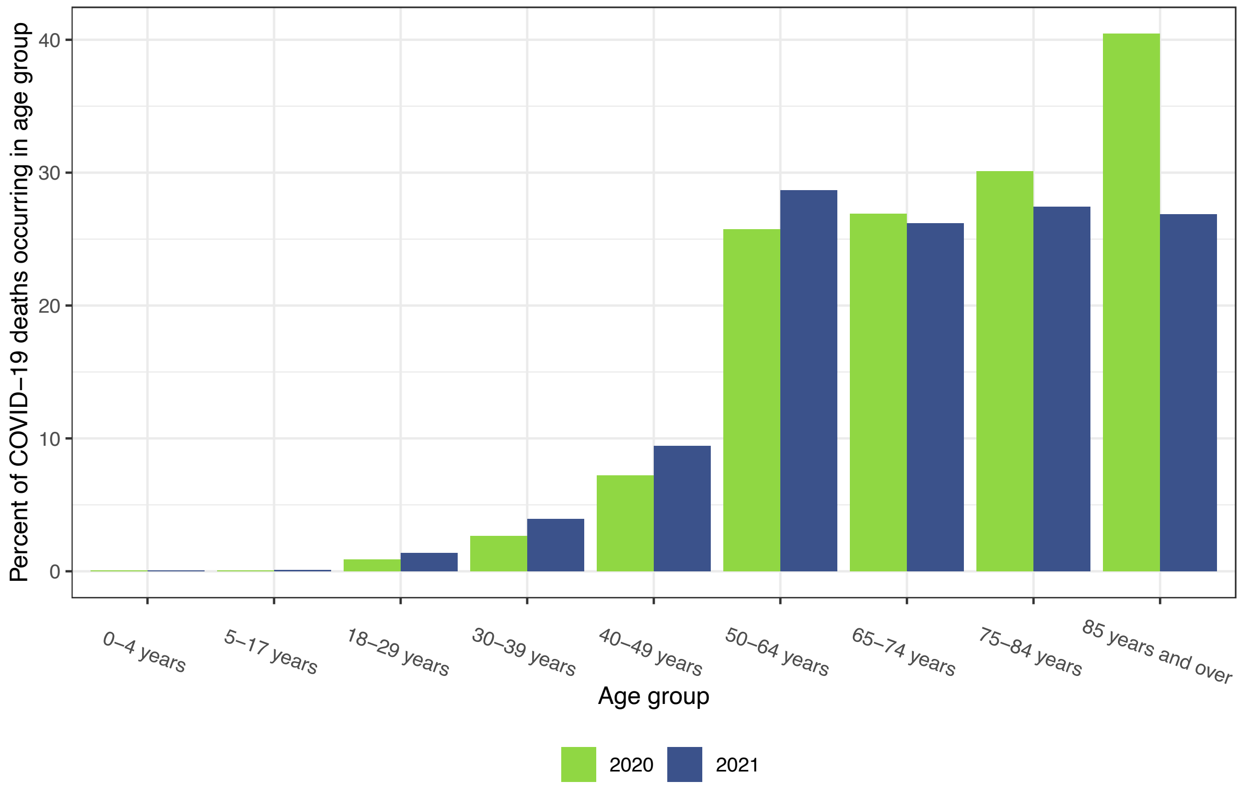

**Figure 1: Percentage of COVID-19 deaths in each age group: 2020 vs. 2021.**
Data are from provisional COVID-19 deaths through October 9, 2021, provided by the National Center for Health Statistics (October 13, 2021 update).

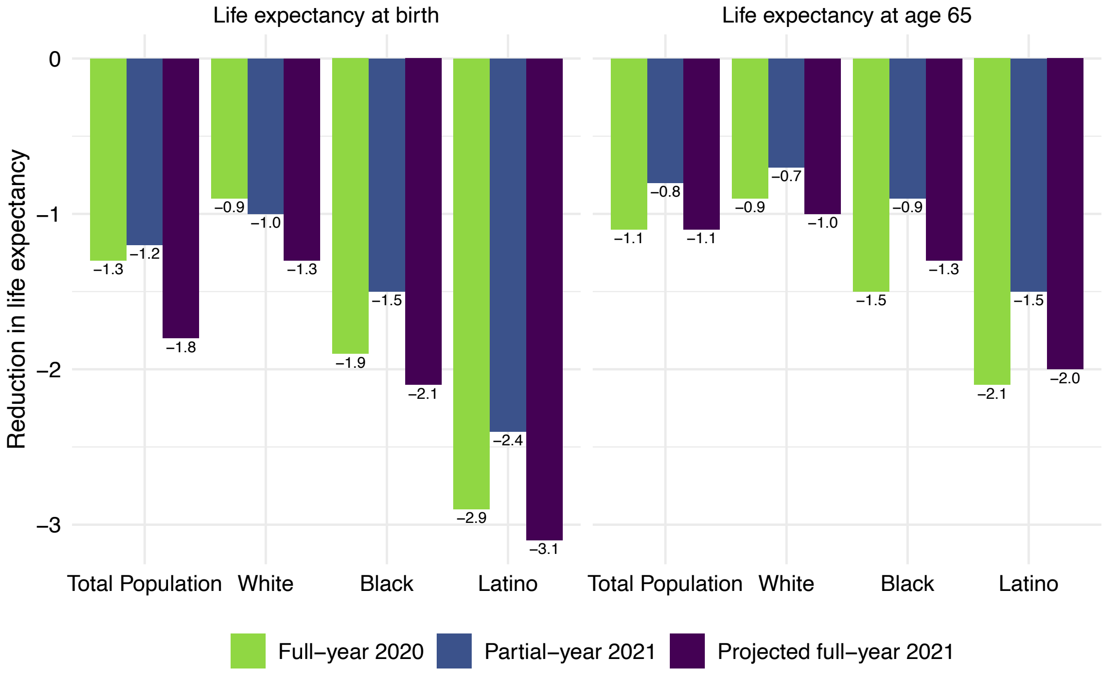

**Figure 2: Reduction in life expectancy at birth due to COVID-19 mortality by race/ethnicity and by year.** Changes are all relative to 2018 life expectancy. 2020 estimates are based on NCHS provisional counts of COVID-19 deaths for all of 2020. 2021 partial-year estimates are based on NCHS provisional counts of COVID-19 deaths through October 9, 2021. 2021 full-year projections are based on the projected number of total reported COVID-19 deaths through December 31, 2021 (October 15, 2021 update).

**Funding**

Research reported in this publication was supported by the National Institute on Aging under Award Number T32AG000037. The content is solely the responsibility of the authors and does not necessarily represent the official views of the National Institutes of Health.
